# Epidemiology of Zoonotic Hepatitis E: A Community-Based Surveillance Study in a Rural Population in China

**DOI:** 10.1371/journal.pone.0087154

**Published:** 2014-01-31

**Authors:** Feng-Cai Zhu, Shou-Jie Huang, Ting Wu, Xue-Feng Zhang, Zhong-Ze Wang, Xing Ai, Qiang Yan, Chang-Lin Yang, Jia-Ping Cai, Han-Min Jiang, Yi-Jun Wang, Mun-Hon Ng, Jun Zhang, Ning-Shao Xia

**Affiliations:** 1 Jiangsu Provincial Center for Disease Control and Prevention, Nanjing, Jiangsu Province, China; 2 National Institute of Diagnostics and Vaccine Development in Infectious Diseases, School of Public Health, Xiamen University, Xiamen, China; 3 Dongtai Center for Disease Control and Prevention, Dongtai, Jiangsu Province, China; Duke University, United States of America

## Abstract

**Background:**

Hepatitis E is caused by two viral genotype groups: human types and zoonotic types. Current understanding of the epidemiology of the zoonotic hepatitis E disease is founded largely on hospital-based studies.

**Methods:**

The epidemiology of hepatitis E was investigated in a community-based surveillance study conducted over one year in a rural city in eastern China with a registered population of 400,162.

**Results:**

The seroprevalence of hepatitis E in the cohort was 38%. The incidence of hepatitis E was 2.8/10,000 person-years. Totally 93.5% of the infections were attributed to genotype 4 and the rest, to genotype 1. Hepatitis E accounted for 28.4% (102/359) of the acute hepatitis cases and 68.9% (102/148) of the acute viral hepatitis cases in this area of China. The disease occurred sporadically with a higher prevalence during the cold season and in men, with the male-to-female ratio of 3∶1. Additionally, the incidence of hepatitis E increased with age. Hepatitis B virus carriers have an increased risk of contracting hepatitis E than the general population (OR = 2.5, 95%CI 1.5–4.2). Pre-existing immunity to hepatitis E lowered the risk (relative risk  = 0.34, 95% CI 0.21–0.55) and reduced the severity of the disease.

**Conclusions:**

Hepatitis E in the rural population of China is essentially that of a zoonosis due to the genotype 4 virus, the epidemiology of which is similar to that due to the other zoonotic genotype 3 virus.

## Introduction

Hepatitis E virus (HEV) is an important public health concern.[1] HEV-infected persons exhibit a wide clinical spectrum, ranging from an asymptomatic infection to fulminant hepatitis.[2] Hepatitis E is usually self-limiting, but chronicity has been associated with organ transplantation and immunosuppression.[3] The high morbidity and mortality among pregnant women and the high infection rates among young children are hallmarks of waterborne outbreaks.[4], [5] The disease is also more severe among people with chronic liver disease.[6], [7]

The virus associated with human disease is divided into four major genotypes[8] and one serotype.[9] There are two distinct epidemiological patterns that correspond to two major viral genotype groups with different host ranges. Genotypes 1 and 2 are human viruses that have been isolated solely from infected humans and account for the epidemiological pattern in most of the developing regions of the world; these are regions where hepatitis E outbreaks occur frequently and often affect several hundred to several thousand people.[4], [10]–[15] The prevalence of anti-HEV antibodies among adults in these areas ranges from 30% to 80%.[16] The second group includes genotypes 3 and 4, which are zoonotic viruses that are distributed worldwide, are common in domestic and wild pigs and humans, and have been associated with sporadic and limited food-borne outbreaks in developed parts of the world.[9], [17]–[21] The seroprevalence rates range from 3% to 20% in these areas.[22]–[30] In the past 10 years, the epidemiologic pattern in China has shifted from a pattern typical of developing areas to a pattern typical of developed countries.[9]

The current understanding of the epidemiological features of hepatitis E associated with the zoonotic types is limited by the lack of data from community-based prospective studies. Recently, we conducted a randomized, controlled clinical trial of the hepatitis E vaccine, HEV239, in an area endemic with HEV (predominantly genotype 4).[31] The study was conducted in 11 rural townships in eastern China. The present report details of the occurrence of hepatitis E in 10 of the 11 townships, with a combined population size of approximately one-half million. The data were collected by a community-wide hepatitis E surveillance system during the 12 months immediately preceding the trial. The findings afford a comprehensive view of the epidemiology of zoonotic hepatitis E in rural eastern China.

## Methods

### Hepatitis surveillance

A sentinel surveillance system monitoring acute hepatitis was conducted over a 12-month period between 2006 and 2007 in 10 townships of Dongtai City in eastern China; the combined number of registered residents was 400,162. An active hepatitis surveillance system covering all the residents was established, which comprised virtually all the village clinic centers, the township hospitals and public and private clinics in the study area.[31] Acute serum samples were obtained from patients presenting at these centers with hepatitis symptoms, such as fatigue and anorexia, for more than 3 days; the serum samples were tested for alanine aminotransferase (ALT) levels. Those with an abnormal ALT level were followed up, and convalescent serum samples were obtained when possible.

Serum samples were obtained from 14,069 individuals who participated in the vaccine trial in two townships [31] to establish baseline IgG anti-HEV values and the prevalence of hepatitis B virus (HBV) in the general population.

Written informed consent was obtained from each patient or vaccine trial volunteer. Approval of the study by the Independent Ethics Committee was obtained from the Ethics Committee of the Jiangsu Provincial Center for Disease Control and Prevention.

### Differential diagnosis of acute hepatitis

The patient who had elevated serum ALT level of more than 2.5 upper limit of normal (ULN) was diagnosed as an acute hepatitis patient. Serum samples from the acute hepatitis patients were tested for the IgM antibody against the hepatitis A virus (IgM anti-HAV), the IgM antibody against the hepatitis B core protein (IgM anti-HBc), the HBV surface antigen (HBsAg) antibody against the hepatitis C virus (anti-HCV) and IgM and IgG anti-HEV. Some samples were also tested for low-avidity IgG anti-HEV and HEV RNA when necessary. A positive finding for IgM anti-HAV was considered to indicate a diagnosis of hepatitis A, and a positive finding for IgM anti-HBc indicated a diagnosis of acute hepatitis B. The diagnosis of hepatitis E was indicated by a positive finding for at least one of three acute markers—IgM anti-HEV, HEV RNA—and an increase in IgG anti-HEV levels of fourfold or more. Hepatitis E attributed to reinfection was indicated by an increase in IgG anti-HEV levels of fourfold or more and a negative finding for IgM anti-HEV, accompanied by the production of high-avidity IgG anti-HEV.[32]

### Serological tests

All of the assays for serological markers were obtained from the Beijing Wantai Biological Pharmacy Enterprise Co. (Beijing, China). The IgG anti-HEV level was expressed in World Health Organization units (Wu) by comparing the level with an assay reference serum of 16.5 Wu/ml; the assay's detection limit was 0.077 Wu/ml.[31] The IgM anti-HEV level was expressed as a ratio using an S/Co cut-off value, with positive being an S/Co value ≥2.[32] The test for low-avidity IgG anti-HEV was conducted on some serum samples as previously described.[32] Briefly, the serum samples were titrated in parallel in the presence and absence of 5 M urea. The avidity of IgG anti-HEV was expressed as the percent of residual antibody levels determined in the presence of 5 M urea relative to those in the absence of 5 M urea. The presence of low-avidity HEV IgG was considered positive when the residual antibody levels were ≤50% of the control values.

### HEV RNA detection

Serum samples were tested for the presence of HEV RNA using reverse-transcription PCR as previously described.[33] Briefly, the total RNA was extracted from a 200-µl sample with Trizol (Invitrogen). A 150-nt segment of open-reading frame 2 (ORF2) was amplified using the primers E1 (5′-CTGTTTAA(C/T) CTTGCTGACAC-3′, nt6260-6279) and E5 (5′-(A/T)GA(A/G) AGCCAAAGCACATC-3′, nt6568-6551) in the first round of PCR; the primers E2 (5′-GACAGAATTGATTTCGTCG-3′, nt6298-6316) and E4 (5′-TG(C/T)TGGTT (A/G)TC(A/G)TAATCCTG-3, nt6486-6467) were used in the second round. The PCR cycling conditions for both rounds consisted of 35 cycles of denaturation at 94°C for 30 sec, annealing at 53°C for 30 sec, and extension at 72°C for 40 sec. Positive samples were sequenced to determine the genotype.

### Statistical Methods

Student's t-test was used for continuous variables, and a two-sided Fisher's exact test was used for counted variables. The differences were considered significant at *P*<0.05. The statistical analyses were performed using OpenEpi (Open Source Epidemiologic Statistics for Public Health, Version 2.3, www.OpenEpi.com, updated 2009/20/05, accessed 2011/01/08). The seroprevalence of IgG anti-HEV was standardized by sex and age according to demographic data for Dongtai City collected in 2007.

## Results

### Etiology of acute hepatitis

**Figure 1:**
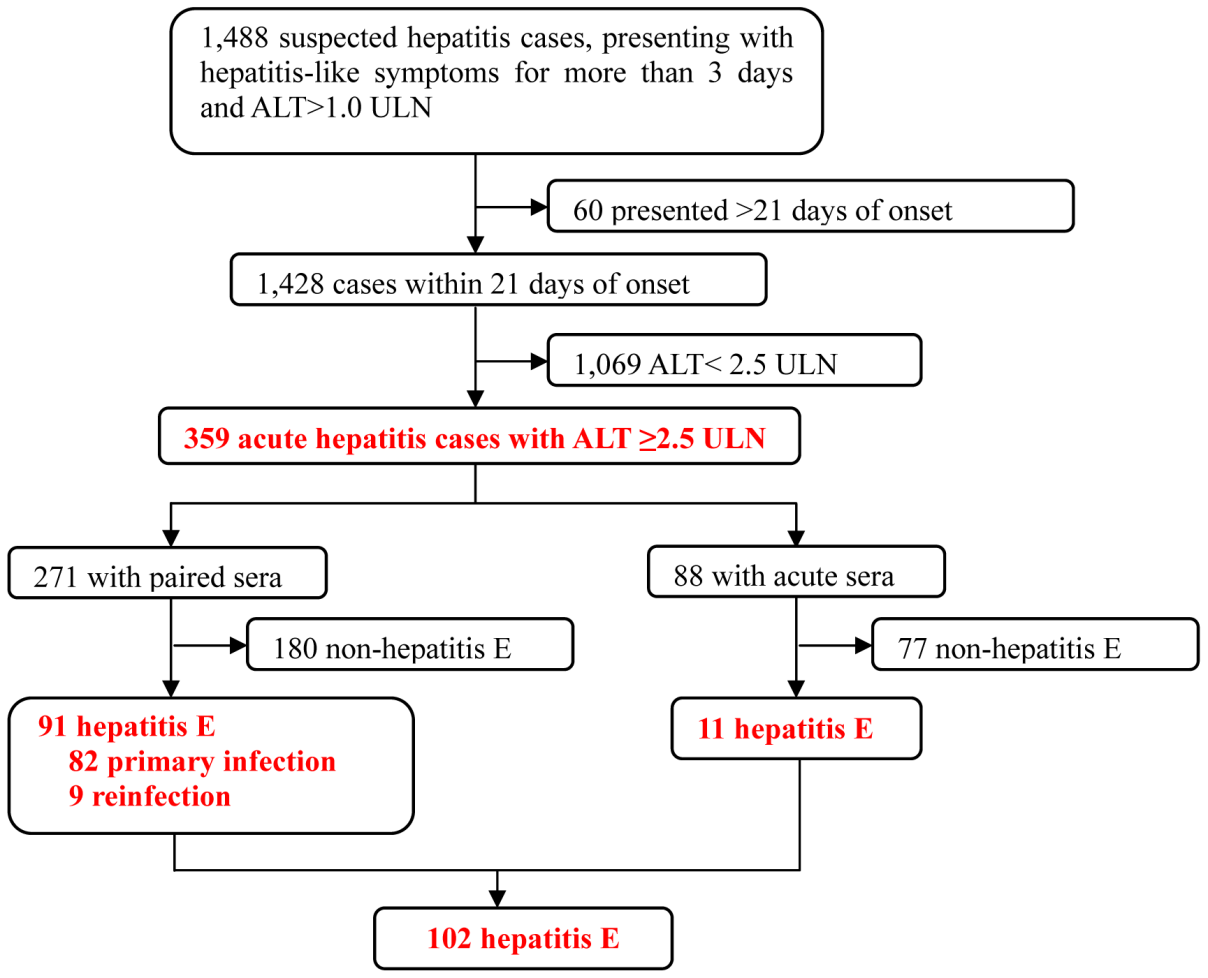
Sentinel surveillance of acute hepatitis in rural community. A sentinel surveillance study of acute hepatitis was conducted over 12 months between 2006 and 2007 in 10 rural townships with a combined population of 400,162. A total of 1,428 suspected cases presented within 21 days of onset of symptoms to local healthcare centers or hospitals during this period and 359 of which were diagnosed with acute hepatitis, having elevated serum ALT levels of 2.5 to 116.7 ULN (Upper Limit of Normal). Paired acute and convalescent serum samples were obtained from 271 of the acute hepatitis patients and single acute samples, from 88 patients for differential diagnosis of acute hepatitis caused by hepatitis viruses.

We have conducted a community-based hepatitis surveillance in 10 rural townships of Dongtai City in eastern China with a combined registered population of 400,162 (Fig 1). The study was conducted over 12 months from October 2006 to September 2007. The standardized HEV anti-IgG prevalence of the study population was 38%. The prevalence of HBsAg was 5.9% (830/14,069). A total of 1,488 suspected hepatitis cases presented with symptoms and constitutional signs during this period and 359 of whom were diagnosed with acute hepatitis (AH), having ALT levels ≥2.5 (2.5 to 116.7) ULN. Of the latter, 148 cases were diagnosed with acute viral hepatitis (AVH), yielding a positive finding for acute markers for HAV (n = 4), HBV (n = 45) or HEV (n = 102), which included 3 patients who were co-infected, or successively infected, with HEV and HBV (Table 1). The other 211 (58.7%) AH cases had an indeterminate diagnosis, yielding a negative finding for acute markers for HAV, HBV and HEV. Hepatitis E accounted for 28.4% (102/359) of the AH cases and 68.9% (102/148) of the AVH cases.

**Table 1:** Etiology of sporadic acute hepatitis.

Differential Diagnosis^a^	N (Ratio)	Ongoing chronic hepatitis virus infection^b^		
		HBsAg	Ig anti-HCV	none
HE	102^c^ (28%)	15	1	86^c^
AHB	45^c^ (13%)	0	0	45^c^
HA	4 (1%)	1	0	3
Indeterminate AH	211 (59%)^d^	80^d^	3^d^	129
total	359^c^	96	4	260^c^

Totally 359 acute hepatitis (AH) patients presented with symptoms of hepatitis and elevated ALT levels ≥2.5 ULN (Fig 1).

a The diagnosis of hepatitis A (HA) was made by positive finding for IgM anti-HAV; the diagnosis of acute hepatitis B (AHB) was made by a positive finding for IgM anti-HBc; and the diagnosis of hepatitis E was made by IgM anti-HEV, HEV RNA and/or ≥4-fold rise of IgG anti-HEV level. The indeterminate AH was indicated by the absence of all the acute viral markers above.

b Underlying chronic infection by HBV was indicated by positive HBsAg and negative IgM anti-HBc. Underlying chronic infection by HCV was indicated by IgG anti-HCV.

c Three of these cases were co-infected with HBV and HEV.

d These cases included one who was chronically infected with both HCV and HBV.

### Epidemiological features of Hepatitis E

**Figure 2:**
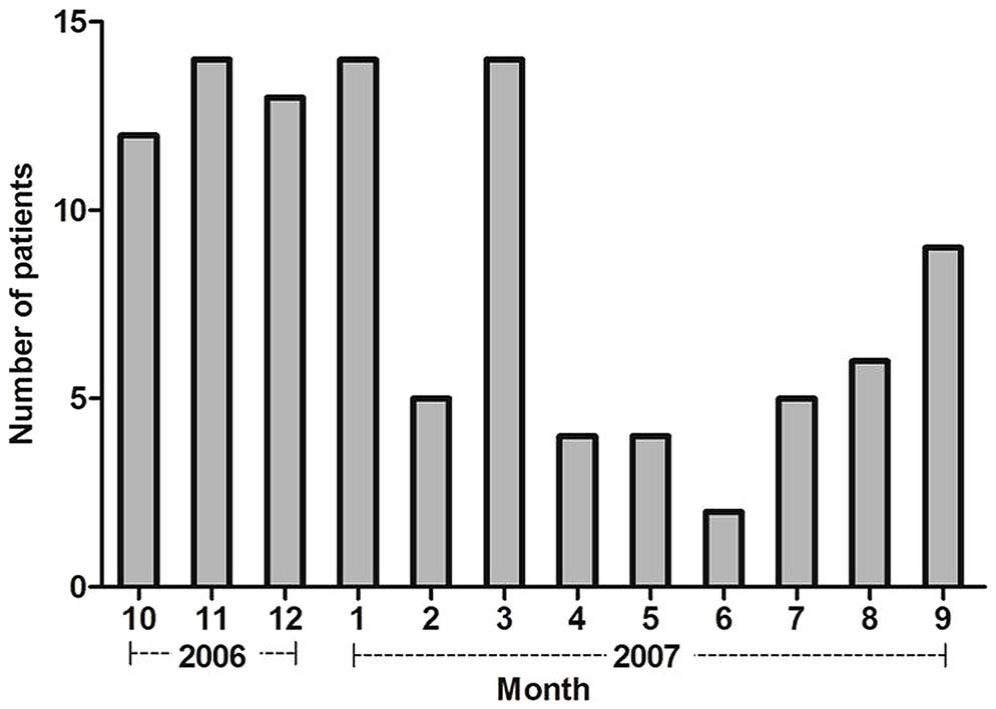
Seasonal distribution of hepatitis E. Hepatitis E cases detected as described in Table 1 occurred sporadically throughout 12 months with ≤2 cases in any one village over any 30 days period. Most (70.6%, 72/102) of the cases occurred in the months of October to March.

**Figure 3:**
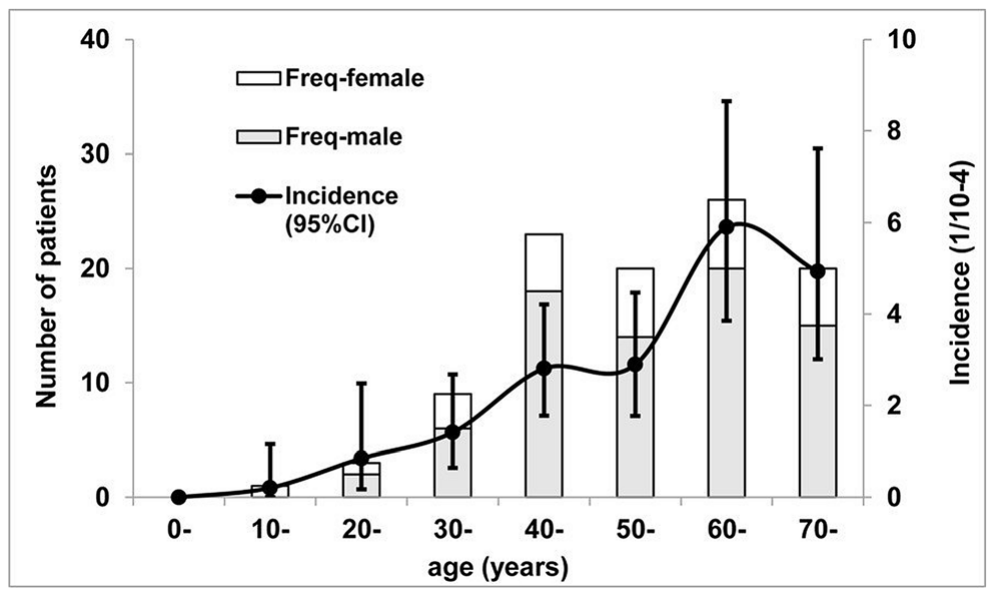
Age and gender distribution of hepatitis E. Noted virtually all the hepatitis E cases (see Table 1) occurred after age 20 years, most of which, among men (shaded block) and few among women (open block). Incidence of the infection (line) increases with age, reaching peak levels after 60 years of age.

Hepatitis E occurred sporadically, with no more than 2 cases detected in the same village within a 30-day period. Infection was more prevalent in the winter and spring (Figure 2). Prevalence of hepatitis E increases with age, male-to-female ratio was 3∶1 and the male preponderance was present at all ages (Fig 3). Of the virus isolates, 93.5% (72/77) were genotype 4 and 6.5% were genotype 1 and no other genotypes were isolated. All of the patients had an uneventful recovery and none among them was pregnant woman.

HE is associated with a greater male preponderance and older ages than acute hepatitis B and indeterminate AH; the peak ALT level of HE patients was also higher and the duration of the illness longer (Table 2). Although HBV carriers have a greater risk of contracting HE (OR = 2.5, 95% CI 1.5 – 4.2), HE patients who are (HBsAg+), or who are not (HBsAg-) HBV carriers have similar age and gender distribution and similar peak ALT levels and duration of illness. Acute hepatitis due to indeterminate causes is associated with substantially lower peak ALT levels and the illness is of shorter duration than AVH. HBV carriage is also associated with higher risk of indeterminate AH (37.9%, 80/211, OR = 6.4, 95% CI 5.1 – 8.1), but does not affect its age and gender distribution or severity of illness.

**Table 2:** Features of sporadic acute hepatitis.

	HE (HBsAg -)	AHB	Indeterminate AH (none)	HE (HBsAg +)	Indeterminate AH (HBsAg +)
Number of cases	83	42	129	15	80
Number (%) of Men	62 (74.7%)	27 (64.3%)	71 (55.0%)*	10 (66.7%)	51 (63.8%)
Mean Age (years)	57.0±13.7	41.2±12.4*	51.7±16.1*	53.5±17.6	41.3±15.7*
Peak ALT (ULN)	16.0±2.6	9.7±2.6*	5.7±2.2*	18.5±2.8	5.1±2.0*
Illness Days	38.7±28.4	16.0±16.4*	17.5±13.2*	39.3±26.4	21.8±20.0*

* Significantly different from HE (*P*<0.05). HE, hepatitis E; AHB, acute hepatitis B; HB, hepatitis B; AH: acute hepatitis; HBsAg +, hepatitis B surface antigen positive. Risk of HE is higher among HBsAg positive than negative subjects (OR = 2.5, 95%CI 1.5- 4.2), while the two groups of patients were of similar gender and age distribution and similar respect to severity of illness.

### Differentiation of Hepatitis E attributed to primary infection and reinfection

**Figure 4:**
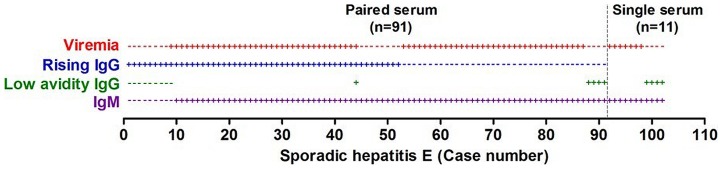
Acute marker profiles of hepatitis E. Of the 102 hepatitis E cases in the present series (see Table 1), 11 (cases 92 to 102) were detected according to occurrence of IgM anti-HEV and HEV RNA in single acute serum samples and the others (cases 1 to 91), according to occurrence of these markers and also ≥4 fold rise of IgG anti-HEV levels in paired serum samples. Additional low avidity IgG anti-HEV test was done for some of the samples, including those tested positive for rising IgG anti-HEV (cases 1 to 8) alone and those positive for IgM anti-HEV alone (cases 88 to 91 and cases 99 to 102). (+) denotes a positive finding and (-), for a negative finding, for the acute marker in individual samples and blank space denotes test not performed. The resulting acute marker profiles of cases 10 to 102 featured a positive IgM anti-HEV, and variously accompanied low avidity IgG or the other acute markers, are consistent with responses to primary infection by hitherto immunologically naïve subjects. The profiles of cases 1 to 9, featuring rising IgG and a negative finding for the other markers, except one (case 9), who was also tested positive for HEV RNA, are consistent with anamnestic responses by immune subjects to reinfection. HEV: hepatitis E virus.

Figure 4 shows the occurrence of three HEV acute markers (HEV RNA, rising IgG anti-HEV levels and IgM anti-HEV) in paired serum samples from 91 hepatitis E patients (cases 1 to 91) and occurrence of two of these markers (HEV RNA and IgM anti-HEV) in single acute serum samples from 11 patients (cases 92 to 102). Additional IgG anti-HEV avidity test was performed for some patients, including those who were positive for rising IgG anti-HEV alone (cases 1 to 8) or IgM anti-HEV alone (cases 88 to 91 and 99 to 102). The acute marker profiles of the 91 patients (cases 10 to 102) feature a positive IgM anti-HEV accompanied by at least one of the other acute markers. These profiles reflect a vigorous IgM response attended by production of low avidity IgG and variously accompanied by rising IgG and/or viremia, which is consistent with responses to primary infection by hitherto immunologically naïve individuals. The acute marker profiles of 9 other patients (case 1 to 9) feature rising IgG anti-HEV levels attended by production of avid IgG anti-HEV, but all yielded a false negative finding for IgM anti-HEV and all, except one (case 9), tested negative for HEV RNA also. Similarly as previously reported cases,[6], [7] such profiles are consistent with anamnestic responses to reinfection by immune subjects.

### Specificity and sensitivity of HEV acute markers

Noted in cases due to primary infection (cases 10 to 102, see Fig 4) that a positive diagnosis by one marker is validated by a positive finding for at least one of the other markers. For example, a diagnosis of hepatitis E by IgM anti-HEV was validated by a positive finding for both rising IgG anti-HEV and HEV RNA, as in cases 10 to 44; by rising IgG anti-HEV, as in cases 45 to 52; by HEV RNA, as in cases 53 to 87 and cases 92 to 98; and by low avidity IgG anti-HEV, as in cases 88 to 91 and cases 99 to 102. This, therefore, confirms specificity of each of the markers for diagnosis of HE, which in turn, validates the definition of hepatitis E adopted for present study.

IgM anti-HEV was found to afford the highest sensitivity of the three markers for diagnosis of HE due to primary infection (cases 10 to 102), while sensitivity of HEV RNA was 82.8% (77/93) and rising IgG anti-HEV was 52.4% (43/82). As previously shown in human [32] and human primates [34], the discrepancy is mainly because viremia subsides and IgG anti-HEV level peaks early during acute phase, whereas IgM anti-HEV is produced early in acute phase and persists until and after the infection resolves. Thus, mean ALT level (7.0±2.3 ULN) of samples taken during convalescence from cases diagnosed by IgM anti-HEV and low avidity IgG anti-HEV (cases 88 to 91 and cases 99 to 102) is significantly lower (*P* = 0.0075) than mean ALT level (17.4±2.5 ULN) for the samples taken at acute phase from the other cases, which were positive for IgM anti-HEV and one or both of the other markers. On the other hand, rising IgG anti-HEV is the only marker available for diagnosis of HE due to reinfection (cases 1 to 9), but false negative rate associating with this marker could not be assessed.

### Incidence and features of hepatitis E

Based on the above and for purpose of estimating incidence of HE, the cases due to primary infection detected using either paired or single serum samples were considered to represent the true number of cases in present series (Table 3). Assuming sensitivity of rising IgG for detecting HE due to reinfection to be the same as for primary infection (52.4%), the true number of cases due to reinfection was expected to be 19.5, 17.2 for those detected using paired samples and 2.3 for those detected using single serum samples. The risk of hepatitis E was estimated to be 2.8 per 10^4^ person-years (py) and the risk of hepatitis E due to primary infection and reinfection was estimated to be 3.8 and 1.3 per 10^4^ py, respectively. While hepatitis E due to primary infection was found to afflict mainly elderly males, those due to reinfection afflict mainly middle age females. Compared with those due to primary infection, hepatitis E due to reinfection provokes a more modest antibody response, the peak ALT levels of the patients were significantly lower, although the duration of illness was similar (Table 3). Although bilirubin was not tested for each serum sample, jaundice was recorded by trained local health team members. The proportion of reported jaundice in primary infection cases (57%, 53/93) was not significantly different from that in reinfection cases (33%, 3/9, *P* = 0.3124). This suggests that pre-existing immunity has lowered risk of hepatitis E (relative risk = 0.34, 95% CI 0.21–0.55) and it appears to have limited the extent of the residual infection that had evaded immune surveillance of affected individuals and, thereby, modulated the antibody response and alleviated severity of the disease caused.

**Table 3:** Features of hepatitis E.

	Total	Primary infection	Reinfection	*P*-value c
Person-years (p-y) at risk a	400,162	247,620	152,542	-
Number of cases observed (expected) b	102 (112.5)	93 (93)	9 (19.5)	-
Based on paired samples	91 (99.2)	82 (82)	9 (17.2)	-
Based on single acute samples	11 (13.3)	11 (11)	0 (2.3)	-
Expected incidence (cases per 10^4^ p-y)	2.8(2.4–3.4)	3.8 (3.0–4.6)	1.3 (0.9–1.9)	<0.0001
Mean age (years)		56.7±13.6	44.2±13.4	0.0104
% Women		21.9% (18/82)	66.7% (6/9)	0.0093
GMC HEV IgG levels (Wu/ml)		80.9±3.0	4.0±3.9	<0.0001
Peak ALT (ULN)		18.7±2.4	6.4±2.0	0.0007
Mean Days of illness		42.1±27.3	40.4±31.1	0.8631

a Person-years (py) at risk for primary infection or for reinfection were calculated based on the estimated exposure rate of 38%, according to the anti-HEV seroprevalence (47%) in a subpopulation of 14,069 subjects in the area, adjusted by sex and age according to the 2007 demographic data of the general inhabitants of the city.

b For the calculation of the expected number of cases (parenthesis), it was assumed that the sensitivity for the detection of hepatitis E attributed to primary infection was 100%, the sensitivity of rising IgG marker for detection of the cases was 52.4% (43/82), and the expected proportion of cases due to reinfection in the series was 17.3% (17.2/99.2).

c Primary infection vs reinfection.

## Discussion

The community-based surveillance described above is a part of a phase III clinical trial of the HEV239 vaccine. [31] It was undertaken to determine disease burden due to HEV. The study was conducted in 10 of 11 townships with a combined population of 400162, which had agreed to participate in that trial, while participation by the rest township was being negotiated. The results arising from this study together with those observed in the following two years [35] by serologic follow-up of control subjects participating in the vaccine trial afford a comprehensive insight to the natural history of HEV infection and disease it causes. The latter study found HEV to be endemic in the study area; infection occurs at 140 per 10^4^ py, virtually all of which was asymptomatic or subclinical and the infection exhibits no significant age or gender bias. [35] Lifelong exposure to the virus had resulted in a seroprevalence of 38%, which cumulates with age.

Presently, incidence of hepatitis E was estimated to be 2.8 per 10^4^ py. It is about 50 times lower than the incidence of infection [35], which is consistent with previous findings showing that HEV is non-cytopathic and avirulent for non human primates [36]. Nevertheless, hepatitis E is a significant disease burden, consisting 28.4% of acute hepatitis and 68.9% of AVH caused by HAV, HBV and HEV combined and accounting for virtually all the enterically transmitted hepatitis, and being the most severe form of acute hepatitis. Essentially a zoonosis, 93.5% of the HE cases were caused by the zoonotic genotype 4, 6.5% by the human genotype 1, and no other genotype was isolated. The disease occurred sporadically throughout the year, more frequently in cold seasons. Whereas HEV infection exhibits no gender or age bias [35], the disease caused affects mainly middle aged and elderly males, suggesting that susceptibility to the disease is significantly a host determined factor. All the patients had an uneventful recovery and none among them was pregnant women. Hepatitis E is more common among HBV carriers than the general population (OR = 2.5, 95%CI 1.5–4.2), but the disease is not more severe among HBV carriers. Hepatitis E was further differentiated according to the occurrence of acute markers in paired samples and estimated that 17.3% of HE is attributed to reinfection and 82.7% to primary infection. The risk of hepatitis E attributed to reinfection (1.3 per 10^4^ py) is 66% lower than primary infection (3.8 per 10^4^ py, RR = 0.34, 95% CI 0.21–0.55), which is similar to the relative risk of reinfection sustained by seropositive subjects [35]. Compared with hepatitis E due to primary infection, disease due to reinfection is milder with lower ALT levels, it afflicts mainly middle aged women and the infection provokes a more modest IgG anti-HEV response. This suggests that pre-existing immunity might have limited the extent of the infection, thereby alleviated severity of the disease caused.

The present study adopted a low ALT limit for the definition of acute hepatitis. This had resulted in a higher proportion of milder form of the indeterminate acute hepatitis in the present series, but it also facilitated detection of the milder form of HE due to reinfection. AVH cause by HAV, HBV or HEV comprised the most severe form of hepatitis in this series. Hepatitis A is rare, partly because of HA vaccine is widely available, and hepatitis E accounted for 68.9% of such cases and for virtually all the enterically transmitted AVH. This testifies to the need to include HEV in routine differential diagnosis of acute hepatitis.

Specificity and sensitivity of the immunological markers used for diagnosis of hepatitis E are traditionally assessed by comparison with the RNA marker in panels of selected serum samples [37]–[42]. Although specific, sensitivity of the RNA marker is not known, however. Consequently, it could not be ascertained whether discrepancies between the HEV RNA and IgM markers is because of false negative on the part of the RNA marker. The bias inherent in the traditional approach was largely overcome in the present study by comparing occurrence of these markers in serum samples taken from consecutive acute hepatitis cases. The discrepancy between the distribution of different acute markers was because of the dynamics the different markers evolve as infection progresses and the nature of the infection itself. Experimental infection of non-human primates showed that viremia subsides and IgG anti-HEV levels peaks early during acute phase, while IgM anti-HEV is produced early in acute phase and persisted until early convalescence [34]. Essentially the same dynamics of marker evolution was observed with serial serum samples taken from the present series of hepatitis E cases[32]. The second main cause for the discrepancy is because the difference between responses to primary infection by hitherto immune naïve host and anamnestic responses mounted by immune hosts. While the response to primary infection features a vigorous IgM antibody response attended by low avidity IgG antibody and variously accompanied by viremia and/or rising IgG antibody level, the response to reinfection features a weak IgM response attended by production of high avidity IgG antibody and transient viremia, and hence is essentially evidenced by rising IgG antibody levels only. In line with this contention, mean ALT levels of the cases positive for IgM and either RNA, rising IgG or both is 17.4±2.5 ULN, which is significantly higher (*P* = 0.0075) than that of 7.0±2.3 ULN for the cases positive for IgM and low avidity IgG, but negative for both of the other markers.

The use of combination of markers allows differentiation of hepatitis E into those due to primary infection and reinfection. For those cases due to primary infection, the different dynamics of marker evolution allows diagnosis by one marker to be validated by other markers. This testifies to the specificity of each of the three markers used in the present study, which in turn validates the definition of hepatitis E adopted for the study. On the other hand, a false negative finding the viral RNA and/or rising IgG antibody levels among these cases is compensated for by a positive finding for IgM antibody. The discrepancy was because the cases were presented at later stages of infection when viremia had subsided and/or IgG antibody levels had peaked, while IgM antibody persisted.

In the present study, exclusion of a diagnosis of hepatitis E is indicated by a negative finding for all three acute markers simultaneously. False negative diagnosis by this approach is considered unlikely, because this would involve a false negative finding to simultaneously involve all the three markers. Thus, the use of combination of markers serves to enhance sensitivity for detection afforded by individual markers. Since the markers are all that are available for diagnosis of hepatitis E, the approach using combination of the markers probably affords the most sensitive means possible for detection of the infection. The false negative rate of this approach could not be ascertained, however, but since a false negative by one marker is compensated for by a positive finding for one or both of the other markers, the number of cases detected by this approach is considered to approximate the true number of such cases, for the purpose of assessing the occurrence of hepatitis E [32].

Genotype 3, the other zoonotic HEV, is widely distributed in developed countries[9]. Seroprevalence of some of which, such as parts of southern France[25] and southwest United Kingdom [28] is comparable to that of the rural eastern and southern China [17], [33]. Recent national surveys in the United States[24] and Denmark[30] revealed that seroprevalence is declining but nevertheless remains significant in the respective countries. Moreover, seroprevalence in the above mentioned countries cumulates with age as does in Dongtai City. Autochthonous cases of hepatitis E reported from these countries are caused by the zoonotic genotype 3 virus and the epidemiology of which is essentially the same as that presently described for that caused by genotype 4 virus.[9] These findings suggest that these countries are endemic for genotype 3 and that the virus is widely distributed, probably in the environment [5], similarly as Dongtai City is endemic for genotype 4 virus. Unlike Dongtai City, however, indigenous cases of hepatitis E are rare in these countries. The inconsistency could be partly because HEV is not included in routine differential diagnosis of acute hepatitis. The inconsistency could also be because, while similarly widely distributed, the environmental viral load in these countries is lower and therefore less likely to result in disease than in Dongtai City. It could also due to genotype 3 is less virulent than genotype 4. Nevertheless, recent studies show that hepatitis E is emerging to be a significant disease burden among immune deficient people [3], [43].

In conclusion, hepatitis E is the major cause of acute viral hepatitis in eastern China. Although the human genotype 1 virus is also prevalent, the epidemiology of hepatitis E in eastern China is essentially that of a zoonosis due to the genotype 4 HEV. It’s outstanding features include: 1) exposure to the virus is universal, but the disease is rare and the likelihood of contracting it increases with age; 2) HEV is the most frequent cause of AVH and the most severe form of HA; 3) the disease occurs sporadically and more frequently in winter and exhibits a marked male preponderance; 4) risk of super-infection in individuals chronically infected with other hepatitis viruses is higher than general population; and 5) naturally acquired immunity does not entirely prevent reinfection, but significantly reduces the risk of infection and alleviates severity of the disease caused.
